# The effects of traditional Chinese manual therapy (Tuina) for chronic fatigue syndrome: A protocol for systematic review and meta-analysis

**DOI:** 10.1097/MD.0000000000027700

**Published:** 2021-11-05

**Authors:** Jun Ren, Tianxiang He, Xin Zhou, Zhiwei Wu, Lingjun Kong

**Affiliations:** a Yueyang Hospital of Integrated Traditional Chinese and Western Medicine, Shanghai University of Traditional Chinese Medicine, Shanghai, China; b Research Institute of Tuina, Shanghai Academy of Traditional Chinese Medicine, Shanghai, China.

**Keywords:** chronic fatigue syndrome, fatigue, Tuina

## Abstract

**Background:**

Chronic fatigue syndrome (CFS) is a common disease and characterized by fatigue, exhaustion, heavy limbs, and dizziness. Tuina, as a traditional Chinese manual therapy, is usually used for CFS in China. Several studies have reported that Tuina can improve fatigue exhaustion, and dizziness of patients with CFS. However, the effects of Tuina for CFS still remain controversial. Therefore, the current systematic review and meta-analysis will be conducted to investigate the effects of Tuina in the management of CFS.

**Methods:**

The comprehensive electronic search of PubMed, Web of Science, Chinese National Knowledge Infrastructure, Wanfang Database, Embase, Cochrane Library, Chinese Science Citation Database, Technology Periodical Database from their inception to October 2021 will be conducted. Randomized controlled trials of Tuina for CFS will be included in the review. Two independent reviewers will complete the study selection, data extraction, and the risk of bias. The meta-analysis will be conducted using the Review Manager Version 5.3 software. The heterogeneity will be assessed using the *I*^*2*^ statistic and Q statistic. The standardized mean difference and 95% confidence intervals will be calculated based on different heterogeneity. The subgroup analysis will be conducted based on the duration of treatment, age, gender, duration of CFS. Quality of evidence will be assessed using the Grades of Recommendation, Assessment, Development and Evaluation.

**Results:**

The current systematic review and meta-analysis will be to investigate the effects of Tuina in the management of CFS.

**Conclusion:**

The conclusion of this study will provide the evidence for the treatment of CFS in the future. It is expected that the conclusions drawn from this review will benefit patients, clinical practitioners and policy makers.

## 1 Introduction

Chronic fatigue syndrome (CFS) is characterized by persistent crippling fatigue for 6 months or more, and usually leads to physical and cognitive difficulties.^[1–3]^ The incidence of CFS in adults is between 0.007% and 2.8%, and it is higher in women, about 4 times higher than in men.^[4]^ It is reported that an estimated 836,000 and 2.5 million Americans suffer from CFS, and the unemployment rate of patients with CFS is between 35% and 69%, and the loss of personal income of patients’ families is about $20,000 a year, causing serious economic losses.^[5]^ The incidence rate of CFS in China is up to 5.58%.^[6]^ Although fatigue is the main manifestation of patients, most patients with CFS have non-restorative sleep disorders, and some patients have musculoskeletal pain, neurocognitive impairment, and emotional disorders.^[7–9]^ Data show that the prevalence rate in the United States increased from 0.0195% to 0.42% in the 10 years from 1989 to 1999.^[6]^ It is precisely because the prevalence rate of CFS is increasing year by year, more and more people suffer from it, which poses a burden on patients’ life and economy, and has become a global challenging disease.

In order to reduce the severity of symptoms and improve functional status, treatments are mainly based on the clinical symptoms of patients with CFS since the cause of CFS has not been clearly defined.^[10,11]^ Although patients with CFS can choose to take drugs to relieve fatigue, the trial shows that except for poor efficacy, uncertainty and inconsistency, each drug therapy lacks epidemiological significance and cannot become the gold standard for CFS treatment.^[12]^ In addition, in the course of drug treatment, patients are often misdiagnosed as severe depression, generalized anxiety disorder, or panic disorder and receive antidepressants and antianxiety drugs.^[13]^ And the efficacy of antidepressants in patients with CFS is very poor.^[14]^ The resistance and dependence of drug therapy also are thorny problems. Therefore, more and more patients with CFS choose complementary and alternative therapies to relieve symptoms such as fatigue. In the world, some CFS patients choose cognitive-behavioral therapy, graded exercise therapy.^[15,16]^ In China, Tuina, as a traditional Chinese manual therapy, is widely used in the management of CFS. Doctors use specific manipulation techniques to act on the patient's skin surface, specific acupoints, or pain and discomfort sites, to improve fatigue of patients with CFS.^[17,18]^ Several studies have reported that Tuina can alleviate the fatigue of patients with CFS and improve the sleep and quality of life.^[19,20]^ However, the effects of Tuina for CFS still remains controversial. There is no systematic review to evaluate the effects of Tuina in the management of CFS.

Therefore, the current systematic review and meta-analysis will be to investigate the effects of Tuina in the management of CFS. The primary outcomes will be focused on fatigue and quality of life of patients with CFS.

## 2 Methods

### 2.1 Study registration

The protocol of this systematic review was conducted by the guideline of Preferred Reporting Items for Systematic Review and Meta-Analysis Protocols 2015.^[21]^ It was registered on the international platform of registered systematic review and meta-analysis protocols (the registration number: INPLASY2021100025) on October 8, 2021.

No ethical statement will be required for the performance of this review and meta-analysis. Results of the study will be disseminated through peer-reviewed journals and conference reports.

### 2.2 Eligibility criteria

#### 2.2.1 Types of studies

In the meta-analysis, only randomized controlled trials of Tuina in the management of CFS will be included. The language will be limited to English and Chinese.

#### 2.2.2 Types of participants

Participants diagnosed as CFS met the diagnostic criteria of CFS revised by the Centers for Disease Control and Prevention in 1994,^[3]^ International Consensus Criteria for ME.^[22]^ There will be no restrictions on gender, education, and race.

#### 2.2.3 Intervention and comparisons

In treatment group, the patients with CFS were treated by Tuina therapy. In control group, the patients received medicine, observation, education, sham manual therapy, and so on.

#### 2.2.4 Outcomes

The primary outcome is assessed by the following fatigue outcomes scales: Fatigue Rating Scale, Fatigue Scale-14, Brief Fatigue Inventory, Fatigue Severity Scale, and Fatigue Impact Scale.

The secondary outcomes will include quality of life, pain, and anxiety. These items will be also evaluated by recognized scales: Short Form-36 Health Survey, Somatic and Psychological Health Report, the Self-Rating Anxiety Scale.

### 2.3 Search strategy

The comprehensive electronic search of PubMed, Web of Science, Chinese National Knowledge Infrastructure, Wanfang Database, Embase, Cochrane Library, Chinese Science Citation Database, Technology Periodical Database from their inception to October 2021 will be conducted by 2 independent reviewers. The search terms include “chronic fatigue syndrome”; “Tuina” or “Chinese massage” or “manual therapy” or “spinal manipulative therapy” or “osteopathy” or “chiropractic”; “frustration” or “weakness” or “tiredness” or “tired”. The search strategy details for PubMed are presented in Table 1. The similar terms will be translated into Chinese for Chinese databases. The language will be limited to English and Chinese.

**Table 1:** Search strategy for PubMed database.

No.	Search items
#1	“Chinese massage” [tiab] OR “Tuina” [tiab] OR “manual therapy” [tiab] OR “osteopathy” [tiab] OR “chiropractic” [tiab] OR “spinal manipulative therapy” [tiab]
#2	“chronic fatigue syndrome” [tiab] OR “fatigue” [tiab] OR “frustration” [tiab] OR “weakness” [tiab] OR “tiredness” [tiab] OR “tired” [tiab]
#3	“Randomized Controlled Trial” [Article type]
#4	#1 AND #2 AND #3

### 2.4 Study collection

**Figure 1:**
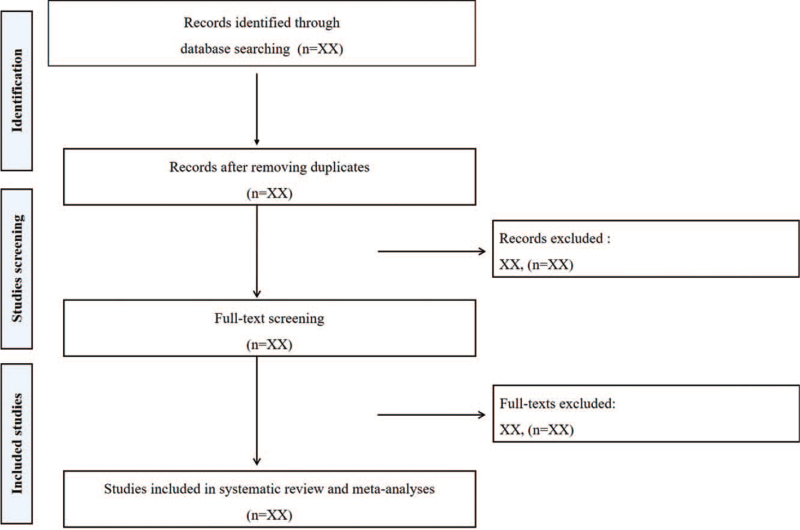
Study selection process.

When 2 independent reviewers conduct literature screening according to the above criteria, they first read the title and abstract of the article. After that, the indicators to be observed are further extracted through the full text. The whole study selection process is shown in Figure 1. Any discrepancies will be resolved by discussion between reviewers.

### 2.5 Data extraction

The data will be extracted by 2 reviewers from the included studies, such as the author information, year of publication, randomization, inclusion and exclusion criteria, the participants information (number of sample, average age, gender, and characteristics of CFS), interventions (types of Tuina therapies, control interventions, time of intervention, outcomes, total treatment duration, curative effect and follow-up time), and adverse events, etc. All reviewers must receive training to guarantee a detailed understanding of the background and objective of the review. Disagreements will be resolved through discussion between reviewers.

### 2.6 Risk of bias assessment

Two reviewers will use the Cochrane Risk of Bias Tool for randomized controlled trial to evaluate the qualified researches. Selection bias, detection bias, attrition bias, performance bias, reporting bias, and other biases will be assessed which will be measured by “low risk”, “high risk” or “unclear”. Reviewers will resolve disagreements through discussion.

### 2.7 Data synthesis

The meta-analysis will be conducted using the Review Manager Version 5.3 software (The Nordic Cochrane Centre, Copenhagen, Denmark). The heterogeneity will be assessed using the *I*^*2*^ statistic and Q statistic. The standardized mean difference and 95% confidence intervals will be calculated based on different heterogeneity. A random-effects model will be carried out When *I*^*2*^-values < 50%, *P* value > .05, there will not be heterogeneity among the studies. If there be heterogeneity among the studies, *I*^*2*^-values > 50%, *P* value < .05.

#### 2.7.1 Subgroup analysis

The subgroup analysis will be conducted based on the duration of treatment, age, gender, duration of CFS.

#### 2.7.2 Sensitivity analysis

Sensitivity analysis will be used to evaluate the reliability of the synthesis results of the meta-analysis of each result index. In case of all included studies have a high risk of bias, sensitivity analyses will be canceled.

#### 2.7.3 Grading the quality of evidence

Quality of evidence will be assessed using the grades of recommendation, assessment, development and evaluation. The result of the assessment will be cross-checked.

## 3 Discussion

With the continuous development of medicine, more and more people pay attention to CFS. Long-term weakness, fatigue, body pain, and insomnia caused by CFS threaten the quality of daily life of patients, but the cause is still unclear. Although there is no description and record of CFS in traditional Chinese medicine, it is classified into the category of deficiency in traditional Chinese medicine according to its symptoms and symptoms.^[23]^ Tuina can relieve the fatigue of CFS patients to some extent.^[24]^ Although there are more and more researches on traditional Chinese medicine therapy in the treatment of CFS in recent years, there is not enough evidence to prove that Tuina is effective in the treatment of CFS. This systematic review and meta-analysis will provide a relatively convincing conclusion as to whether Tuina can effectively treat fatigue symptoms in patients with CFS. The conclusions of this review may provide evidence-based advice for the health care system, optimize clinicians’ treatment strategies, and find promising Tuina prescriptions for researchers.

## Author contributions

**Conceptualization:** LK, JR, TH.

**Funding acquisition:** LK, TH.

**Methodology:** JR, TH, XZ.

**Project administration:** XZ, ZW.

**Writing – original draft:** JR, TH, XZ.

**Writing – review & editing:** LK, ZW.
